# Multiomics analysis of canine myocardium after circumferential pulmonary vein ablation: Effect of neuropeptide Y on long‐term reinduction of atrial fibrillation

**DOI:** 10.1111/jcmm.18582

**Published:** 2024-08-06

**Authors:** Qiyuan Song, Ning Zhang, Yujiao Zhang, An Zhang, Huilin Li, Shuting Bai, Luxiang Shang, Juanjuan Du, Yinglong Hou

**Affiliations:** ^1^ Department of Cardiology The First Affiliated Hospital of Shandong First Medical University & Shandong Provincial Qianfoshan Hospital, Shandong Medicine and Health Key Laboratory of Cardiac Electrophysiology and Arrhythmia, Shandong First Medical University Jinan China; ^2^ Medical Integration and Practice Center, Shandong University Jinan China; ^3^ Peking University Beijing China; ^4^ Department of Emergency Medicine The First Affiliated Hospital of Shandong First Medical University & Shandong Provincial Qianfoshan Hospital, Shandong Medicine and Health Key Laboratory of Emergency Medicine Jinan China

**Keywords:** atrial fibrillation, catheter ablation, NPY, recurrence

## Abstract

Catheter ablation (CA) is an essential method for the interventional treatment of atrial fibrillation (AF), and it is very important to reduce long‐term recurrence after CA. The mechanism of recurrence after CA is still unclear. We established a long‐term model of beagle canines after circumferential pulmonary vein ablation (CPVA). The transcriptome and proteome were obtained using high‐throughput sequencing and TMT‐tagged LC‐MS/LC analysis, respectively. Differentially expressed genes and proteins were screened and enriched, and the effect of fibrosis was found and verified in tissues. A downregulated protein, neuropeptide Y (NPY), was selected for validation and the results suggest that NPY may play a role in the long‐term reinduction of AF after CPVA. Then, the molecular mechanism of NPY was further investigated. The results showed that the atrial effective refractory period (AERP) was shortened and fibrosis was increased after CPVA. Atrial myocyte apoptosis was alleviated by NPY intervention, and Akt activation was inhibited in cardiac fibroblasts. These results suggest that long‐term suppression of NPY after CPVA may lead to induction of AF through promoting cardiomyocyte apoptosis and activating the Akt pathway in cardiac fibroblasts, which may make AF more likely to reinduce.

## 1 INTRODUCTION

Atrial fibrillation (AF) is the most common tachyarrhythmia clinically, affecting approximately 370,000 adults worldwide.^1^, ^2^ Serious complications such as stroke, heart failure, cognitive impairment and cardiac arrest and increasing health care costs of AF^3^, ^4^ seriously affect the quality of life of patients, so its treatment has become a major clinical challenge. Catheter ablation (CA) of AF is an important part of the rhythm control strategy. As a first‐line treatment for AF, it has been shown to be superior to antiarrhythmic drugs in refractory AF and is widely used in clinical treatment.^5^, ^6^, ^7^ However, the high recurrence rate of AF after CA limits its effectiveness. Despite significant advances in ablation techniques and clinical practice, the long‐term recurrence rate of AF has not been effectively controlled, and up to 35% of patients require repeat ablation.^8^ Although many studies have investigated the recurrence of AF, the mechanism of recurrence has not been fully elucidated.

The pulmonary vein (PV)‐left atrium (LA) junction has been shown to be an important source of abnormal foci in AF.^9^, ^10^ Therefore, pulmonary vein isolation (PVI) has become the basis for CA of AF.^11^, ^12^ The PV‐LA reconnection and extra‐PV triggering have been proved to be the main mechanisms of AF recurrence after ablation. However, studies^13^ indicate that even in patients without AF recurrence, 58% of cases exhibited the restoration of PV electrical conduction. Furthermore, even after additional ablation of non‐PV triggers following PVI, there is still a recurrence rate of 22%–29%.^14^ The mechanisms underlying AF recurrence are still subject to further investigation. It has been demonstrated that the main nerve plexus of atrium is distributed in the antrum and ostium of PV, which is the most densely distributed part of nerve tissue.^9^, ^15^ Therefore, CPVA could have a denervation effect in addition to electrical isolation of PVs and may cause neural remodelling of local target tissue.^8^ Our previous study^16^ showed long‐term nerve remodelling after denervation was related to AF reinduction. Our another study^17^ also found that significant abnormal changes in the ultrastructure of target atrial myocytes occurred 1 month after ablation, which persisted up to 12 months after ablation. Meanwhile, myocardial interstitial collagen deposition increased with time, suggesting that denervation may be an important factor leading to myocardial fibrosis.^17^

Neuropeptide Y is a 36‐amino acid polypeptide that acts as a neurotransmitter or neuromodulator and is the most abundant neuropeptide in the central and peripheral nervous system as well as in the heart.^18^ The mechanism of NPY in AF and AF recurrence has not been elucidated. Therefore, in this study, based on the long‐term model of circumferential pulmonary vein ablation in canines, we further studied the mechanism of long‐term reinduction of AF and the role of NPY in the reinduction.

## 2 METHODS

### 2.1 Ethics approval statement

All experimental protocols were approved by the Ethics Committee of the First Affiliated Hospital of Shandong First Medical University according to the NIH Guide for the Care and Use of Laboratory Animals.

### 2.2 Construction of the CPVA model in beagle canines

**FIGURE 1:**
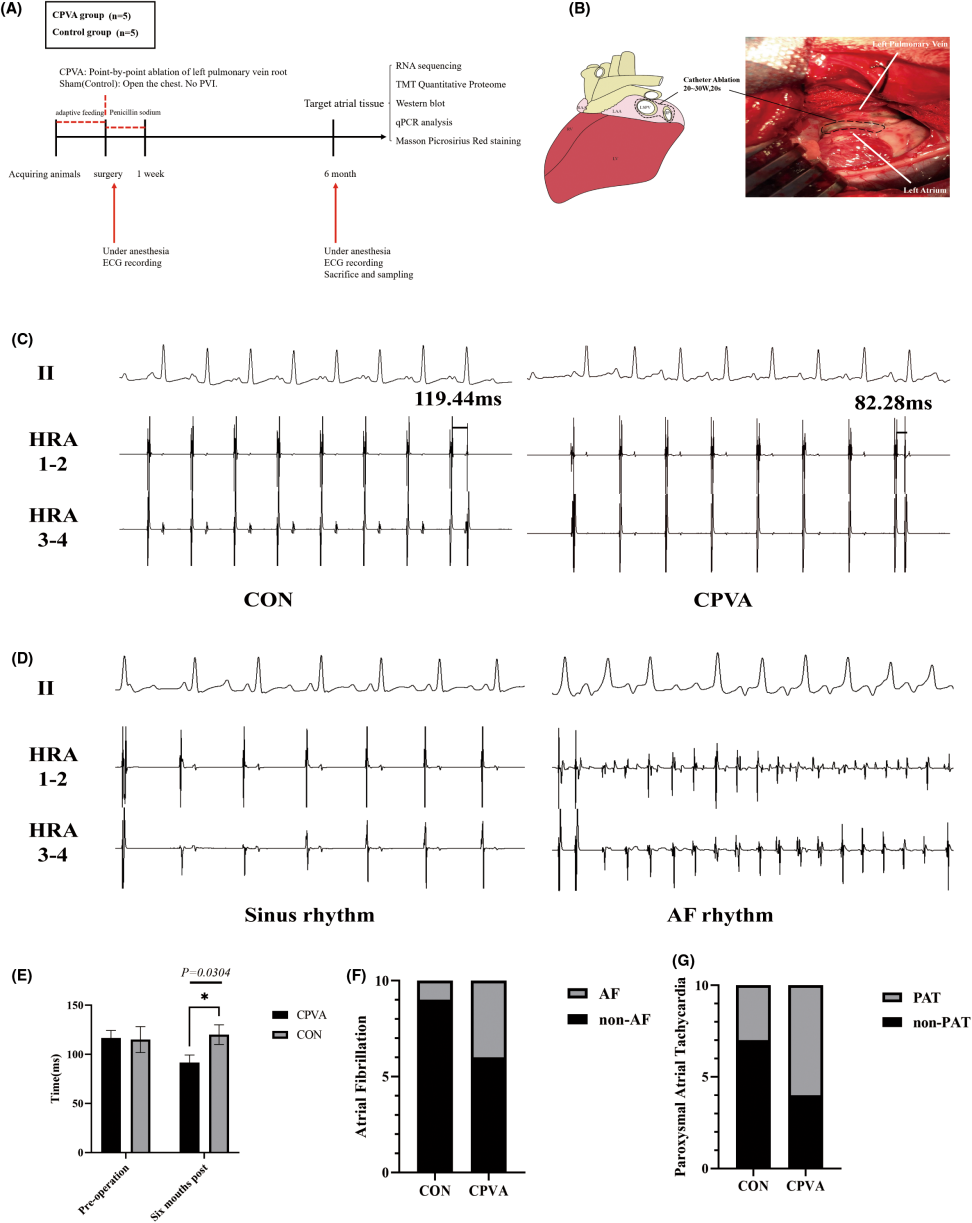
(A) The CPVA model is processed at different times. (B) CPVA schematic. (C) At 6 months, the characteristics of electrophysiological changes in the control group and CPVA group (D) The electrocardiogram of SR and AF. (E) Changes in left atrial AERP preoperatively and at 6 months (*p < 0.05). (F) Inducibility of AF in CPVA versus CON at 6 months. (G) Inducibility of PAT in CPVA versus CON at 6 months. SR, sinus rhythm; PAT, paroxysmal atrial tachycardia.

Ten beagle canines weighing 10–12 kg, male or female, were randomly divided into 2 groups: the CPVA group (*n* = 5), in which right fourth intercostal thoracotomy was performed, and then CPVA was performed; and the sham operation group (control group) (*n* = 5), in which thoracotomy was performed in the right fourth intercostal space without CPVA. Both groups were observed for 6 months after the operation (Figure 1A).

In the CPVA group, the radiofrequency current was delivered to the left PV region using a 7Fr deflectable quaternary catheter with a 4 mm tip electrode. The catheter tip was positioned on the surface of the left PV root under direct visual control to ensure optimal tissue contact and energy delivery, and ablation was performed point‐by‐point along the left PV root (Figure 1B). The ablation endpoint was defined as the continuous ablation line observed directly below the root of the left PV.

Electrophysiological examinations were completed 6 months after CPVA, and the animals were sacrificed by air embolism following ethical system guidelines. All samples were obtained from the target atrial tissue (1 cm radius from the ablation line) and stored at −80°C for subsequent experiments. Some specimens were fixed with 4% paraformaldehyde (Servicebio, Wuhan, China) followed by Masson staining and picrosirius red staining.

### 2.3 In vivo electrophysiological testing

Three bipolar electrodes were placed in the left atrial free wall proximal to the left PV and in the high LA and left atrial appendage distal to the left PV. Pacing leads were removed after the initial study (baseline) and replaced during the final experiment (6 months postprocedure). The drive chain consists of 8 stimuli with a basic cycle length of 350 ms followed by a premature extra stimulus (S2) with a coupling interval of 200 ms and a step size of 5 ms. The atrial effective refractory period (AERP) was defined as the longest S1 to S2 interval that failed to produce a diffuse response. Data were collected for each animal prior to ablation and at 6 months postablation. AERP was measured immediately before CPVA and 6 months after CPVA.^16^ Programmed electrical stimulation with burst stimulation (120 ms) was used to induce AF, and a rapid irregular atrial rhythm lasting longer than 10 s was set as successful induction, while a rhythm of less than 10 s was defined as paroxysmal atrial tachycardia (PAT).

### 2.4 High‐throughput RNA‐Seq

(1) Total RNA was extracted from target atrial muscle tissue, 1 mL TRIzol (Magen (R4801‐02)) was added to the sample ground in liquid nitrogen, and total RNA was extracted by TRIzol method. Then, the sample concentration and 260/280, 260/230 ratio were measured and recorded by Nanodrop (ThermoFisher (ND‐2000)), and the integrity of RNA was checked by Agilent 4150 (Agilent Technologies Inc.). (2) For the library construction, oligo (DT) beads were used to enrich eukaryotic mRNA, fragmentation buffer was added to randomly interrupt mRNA, mRNA was used as template to synthesise two strands of cDNA, and double‐stranded cDNA was purified by AMPure XP beads. The purified double‐stranded cDNAs were end‐repaired and A‐tailed, and then the fragment size was selected by AMPure XP beads. Finally, the final cDNA library was obtained by PCR enrichment. (3) For quality control and sequencing, the length and effective concentration of the insert fragment of the library was determined, and then pooling was performed according to the target off‐machine data amount and on‐machine sequencing.

### 2.5 TMT labelling and high PH reversed‐phase fractionation

Labelled peptides were fractionated by the High pH Reversed‐Phase Peptide Fractionation Kit (Thermo Scientific). The dried peptide mixture was reconstituted, acidified with 0.1% TFA solution, and loaded onto the equilibrated, high‐pH, reversed‐phase fractionation spin column. Peptides were bound to the hydrophobic resin under aqueous conditions and desalted by washing the column with water by low‐speed centrifugation. A step gradient of increasing acetonitrile concentrations in a volatile high‐pH elution solution was then applied to the columns to elute bound peptides into 10 different fractions collected by centrifugation. The collected fractions were desalted on C18 cartridges (Empore™ SPE Cartridges C18 (standard density), bed I.D. 7 mm, volume 3 mL, Sigma) and concentrated by vacuum centrifugation.

### 2.6 LC‐MS/MS analysis

LC‐MS/MS analysis was performed using a mass spectrometer (Thermo Scientific) with peptides loaded onto a reversed‐phase trap column (Thermo Scientific Acclaim PepMap100) connected to a C18 reversed‐phase analytical column (Thermo Scientific Easy Column). It was then separated using 0.1% formic acid and eluted with a linear gradient of 84% acetonitrile and 0.1% formic acid at 300 nL/min controlled by IntelliFlow technology. The mass spectrometer was operated in positive ion mode. MS data were acquired using the data‐dependent top10 method, with HCD fragmentation based on the most abundant precursor ion in the survey scan (300–1800 m/z).

### 2.7 Data processing

Differential expression in transcriptomics was investigated using the DESeq2 package in R software, and genes were defined as differentially expressed genes (DEGs) after CPVA when |log_2_ Fold Change| ≥1, corrected *p* < 0.05. The differential expression of proteins was analysed using the *T* test, and proteins were defined as differentially expressed proteins (DEPs) when |log_2_ Fold Change| ≥0.26 and *p* < 0.05.

### 2.8 Enrichment analysis

Gene Ontology (GO) annotation and Kyoto Encyclopedia of Genes and Genomes (KEGG) pathway enrichment analysis were performed on the DAVID database (https://david.ncifcrf.gov/). GO and pathway enrichment analyses were performed for DEGs and DEPs, respectively, by using *p* < 0.05 as a threshold.

### 2.9 Isolation of primary rat atrial myocytes

Primary atrial myocytes were isolated from 1‐ to 3‐day‐old SD rats. Atrial tissue was cut into pieces. Then, they were digested in 0.08% EDTA‐free trypsin (Solarbio, Beijing, China) at 4°C overnight, washed with calcium‐ and magnesium‐containing Hanks solution, and digested in 0.08% collagenase type II until the tissue was not visible. The cell suspension was seeded in a culture dish and allowed to adhere for 2 h to isolate primary fibroblasts and primary atrial myocytes, and the primary atrial myocytes were seeded in a new culture plate. Primary atrial myocytes were cultured in high glucose medium containing 10% foetal bovine serum, 1% penicillin–streptomycin and 0.01% bromodeoxyuridine (Sigma, Shanghai, China) for purification of atrial myocytes.

### 2.10 Cell counting kit‐8 (CCK‐8) assay

Cell growth was detected using a Cell Counting Kit‐8 (CCK‐8) assay (Elabscience, Wuhan, China). Rat primary atrial myocytes were seeded into 96‐well plates (5000–8000 cells per well) and cultured overnight. Subsequently, NPY (MCE, Cat. No: HY‐P0198A) treatments were administered at concentrations of 0, 1, 10, 50, 100, 150, 200, 250 and 300 nM. After 48 h of incubation, 10 μL/well of CCK‐8 dilution was added to each well, followed by an additional 2 h of incubation. Absorbance was checked at 450 nm using an ultraviolet spectrophotometer (Molecular Devices, CA, USA).

### 2.11 Flow cytometry

Apoptosis detection was performed using Annexin V‐FITC and PI apoptosis kits (Yeasen Biotechnology, Shanghai, China). Primary rat atrial myocytes were cultured in 6‐well plates and treated with different concentrations of NPY for 48 h. The cells were digested with EDTA‐free trypsin, washed 3 times with PBS and stained with Annexin V‐FITC and PI. The fluorescence emitted by the cells was analysed by flow cytometry to obtain events for each sample using an overall “dot plot”, where each dot corresponds to a single event of a specific fluorescence signal signature of the reference axis. The data were analysed using Flow Jo 10.6.2 software.

### 2.12 Immunofluorescence

Primary rat cardiac fibroblasts were treated with varying concentrations of NPY. They were fixed in 4% paraformaldehyde, incubated with primary antibodies according to the instructions at 4°C overnight and then treated with fluorescent secondary antibodies and incubated at 37°C for 1.5 h. DAPI (Beyotime Biotechnology, Beijing, China) was added and incubated for 5 min at room temperature. The anti‐quencher (Servicebio, Wuhan, China) was added dropwise to the coverslip and observed under a fluorescence microscope. The expression levels of fibronectin (Servicebio, 1:1500, 113,491) and collagen III (Servicebio, 1:250, 11,629) were measured. Statistical analysis.

### 2.13 Western blot

The isolated target atrial muscle tissue was ground by a tissue grinder, or the treated rat primary atrial muscle cells were lysed in a mixture of rapid tissue lysis solution (RIPA, Beyotime Biotechnology, Beijing, China), protease inhibitor (PMSF, Beyotime Biotechnology, Beijing, China) and phosphatase inhibitor (Beyotime Biotechnology, Beijing, China). Supernatants were separated using a high‐speed, and the protein concentration was measured using BCA (Beyotime Biotechnology, Beijing, China). Depending on the measured protein concentration, proteins were loaded onto SDS–PAGE gels and polyvinylidene (PVDF) membranes. The secondary antibody (HRP‐labelled goat anti‐rabbit IgG, Beyotime Biotechnology, Beijing, China; dilution, 1:10,000) for 50 min the next day. The expression levels of β‐actin (CST, 1:1000, 3700), NPY (CST, 1:1000, 11,976), Fibronectin 1 (Abcam, 1:1000, ab268020), Collagen III (Proteintech, 1:500, 22,734‐1‐AP), FAP (Abcam, 1:1000, ab314456), Vimentin (Abcam, 1:1000, ab92547), α‐SMA (Proteintech, 1:500, 55,135‐1‐AP), Bcl2 (Proteintech, 1:1000, 26,593‐1‐AP), Bax (CST, 1:1000, 2772), Collagen I (Abcam, 1:1000, ab260043), TGF β 1 (Abcam, 1:1000, ab215715), p‐Akt Ser473 (CST, 1:2000, 4060) and Akt (CST, 1:1000, 4691) were detected. The grey values of each band were quantified using ImageJ v.1.53a software and normalized to β‐actin.

### 2.14 Real‐time fluorescence quantitative PCR analysis

RNA was extracted from target atrial tissue and primary rat cardiomyocytes by the TRIzol method. First‐strand cDNA was synthesized using a reverse transcription kit (Invitrogen Carlsbad, CA, USA). Relative mRNA expression of the genes was measured by a real‐time quantitative PCR kit (Invitrogen). The PCR primer sequences of each gene were as follows: FORWARD: 5′‐TGCGACACTACATCAACCTCATCAC‐3′ and REVERSE: 5′‐AGAAGGGTCATCCAGCCTAGTTCTG‐3′.

### 2.15 Statistical analysis

Baseline characteristics for statistical analysis were script statistics. All numerical variables are expressed as the mean ± standard deviation. Unpaired two‐tailed Student's *t* test was used to determine the difference between the two groups, while analysis of variance (ANOVA) followed by Bonferroni's post hoc test was applied for multiple comparisons. For groups with only one variable, one‐way ANOVA was used, while two‐way ANOVA was performed for the two categorical independent variables. Categorical variables are expressed as numbers, and percentages were compared for statistical analysis using GraphPad Prism v.9 software.

This paper's Methods section provides essential details about the experiments. Additional experimental methods and further detailed information can be found in the supplementary materials.

## 3 RESULTS

### 3.1 Establishment of the CPVA model and cardiac electrophysiological examination

Ten canines remained stable during the procedure, and subsequent analyses were based on these canines.

Cardiac electrophysiological testing revealed a significant shortening in AERP in the CPVA group 6 months postablation compared to preablation. Specifically, the AERP was shortened from 117 to 92 ms in the CPVA group (Figure 1C). In addition, compared with the control group, the AERP was also significantly shortened in the CPVA group (Figure 1E). The incidence of atrial tachycardia was detected through burst stimulation. As indicated by the results, the incidence of AF and PAT in the CPVA group was higher than the control group (Figure 1F,G).

### 3.2 Transcriptome and proteome analysis

**FIGURE 2:**
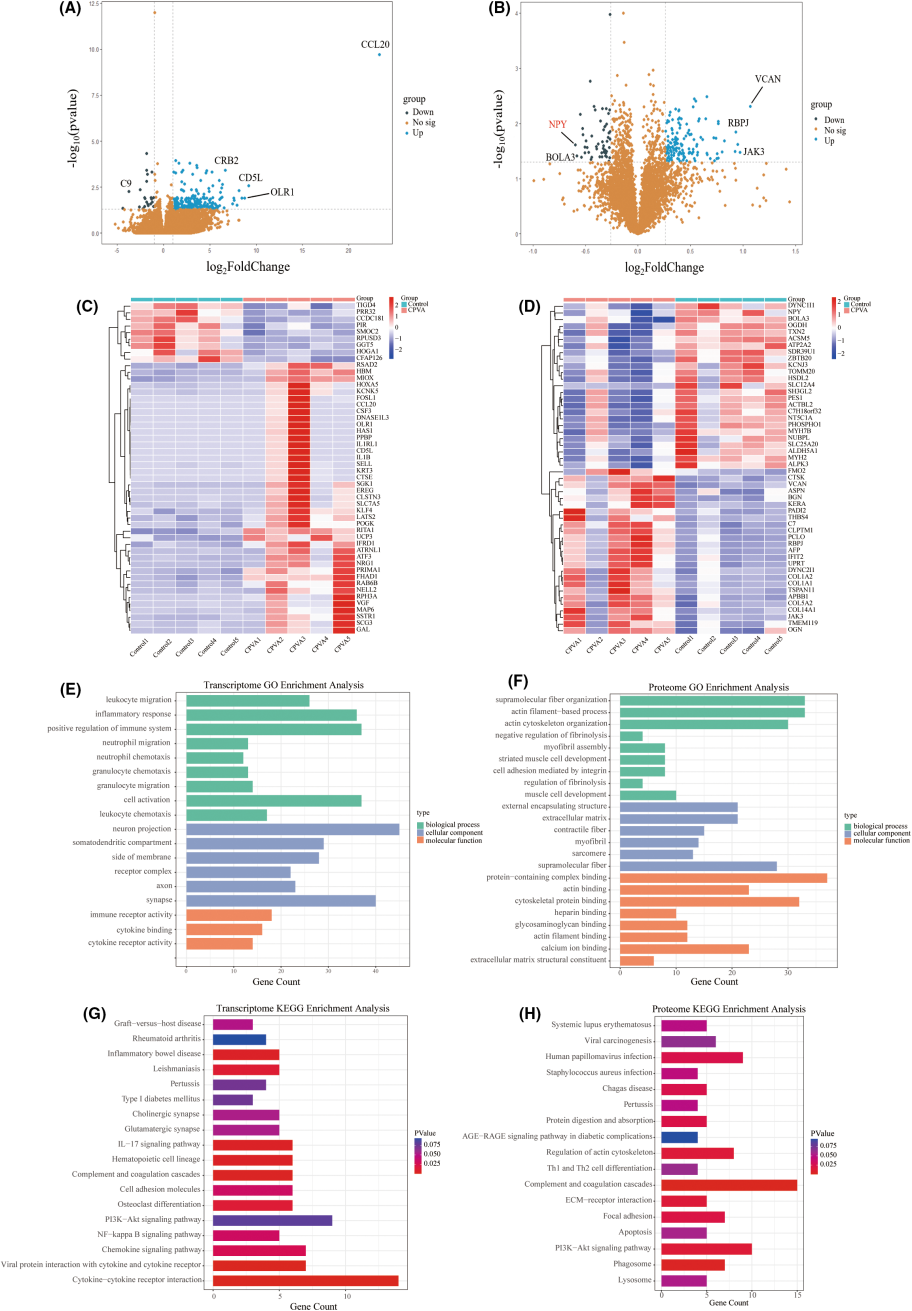
(A) Volcano plot of transcriptome results after CPVA. (B) Volcano plot of proteome results after CPVA. (C) Heatmap analysis of differentially expressed genes after CPVA. (D) Heatmap analysis of differentially expressed proteins after CPVA. (E) GO terms of DEGs after CPVA. (F) GO terms of DEPs after CPVA. (H) KEGG terms of DEGs after CPVA. (G) KEGG terms of DEPs after CPVA. (E) Combination of the pathways enriched in DEGs and DPEs. BP, Biological process; CC, Cellular component, MF, Molecular function. KEGG, Kyoto Encyclopedia of Genes and Genomes; DEGs, Differentially expressed genes; DEPs, Differentially expressed proteins; GO, Gene Ontology.

Thirty‐one thousand eight hundred ninety‐seven genes were detected using high‐throughput sequencing technology; a total of 259 DEGs were screened (237 genes significantly upregulated and 22 genes significantly downregulated). A volcano plot elucidated the variance in DEGs based on *p* value and log_2_FoldChange (Figure 2A). The DEGs are further demonstrated (Figure 2C) (Table S1).

TMT labelling LC‐MS/MS technology detected 4417 proteins, 186 DEPs were screened (134 of which were significantly upregulated and 56 were significantly downregulated). A volcano plot elucidated the variance in DEPs based on *p* value and log_2_FoldChange (Figure 2B). The top DEPs are further demonstrated (Figure 2D; Table S2). We chose the most highly downregulated protein, NPY for further study.

### 3.3 GO and KEGG enrichment analyses

DEGs and DEPs were categorized and annotated by GO functional, which used three structured and controlled vocabularies to describe the gene and protein products in terms of associated biological processes (BPs), cellular compositions (CCs) and molecular functions (MFs). The results showed that the functions of DEGs were mainly involved in the following biological processes: neuronal action potential; transmission of nerve impulse; leukocyte migration; action potential; neutrophil chemotaxis and inflammatory response (Figure 2E). The results showed that the functions of DEPs were mainly involved in the following biological processes: actin filament‐based process; actin cytoskeleton organization; negative regulation of fibrinolysis; myofibril assembly; striated muscle cell development; cell adhesion mediated by integrin; and regulation of fibrinolysis (Figure 2F).

KEGG pathway analysis showed that the DEGs were enriched in the following pathways: cytokine–cytokine receptor interaction; IL‐17 signalling pathway; chemokine signalling pathway; NF‐kappa B signalling pathway; cell adhesion molecules; cholinergic synapse; glutamatergic synapse; and PI3K‐Akt signalling pathway (Figure 2G). KEGG pathway analysis showed that the DEPs were enriched in the following pathways: complement and coagulation cascades; PI3K‐Akt signalling pathway; ECM‐receptor interaction; regulation of actin cytoskeleton; apoptosis; Th1 and Th2 cell differentiation; and AGE‐RAGE signalling pathway in diabetic complications (Figure 2H).

The PI3K‐Akt signalling pathway was enriched in KEGG pathway enrichment analysis of DEGs and DEPs. Thus, we suggest that the activation of PI3K‐Akt signalling pathway may influence the changes in target atrial tissue after CPVA.

### 3.4 Neuropeptide Y was decreased and fibrosis‐associated protein was increased in target atrial muscle after CPVA

**FIGURE 3:**
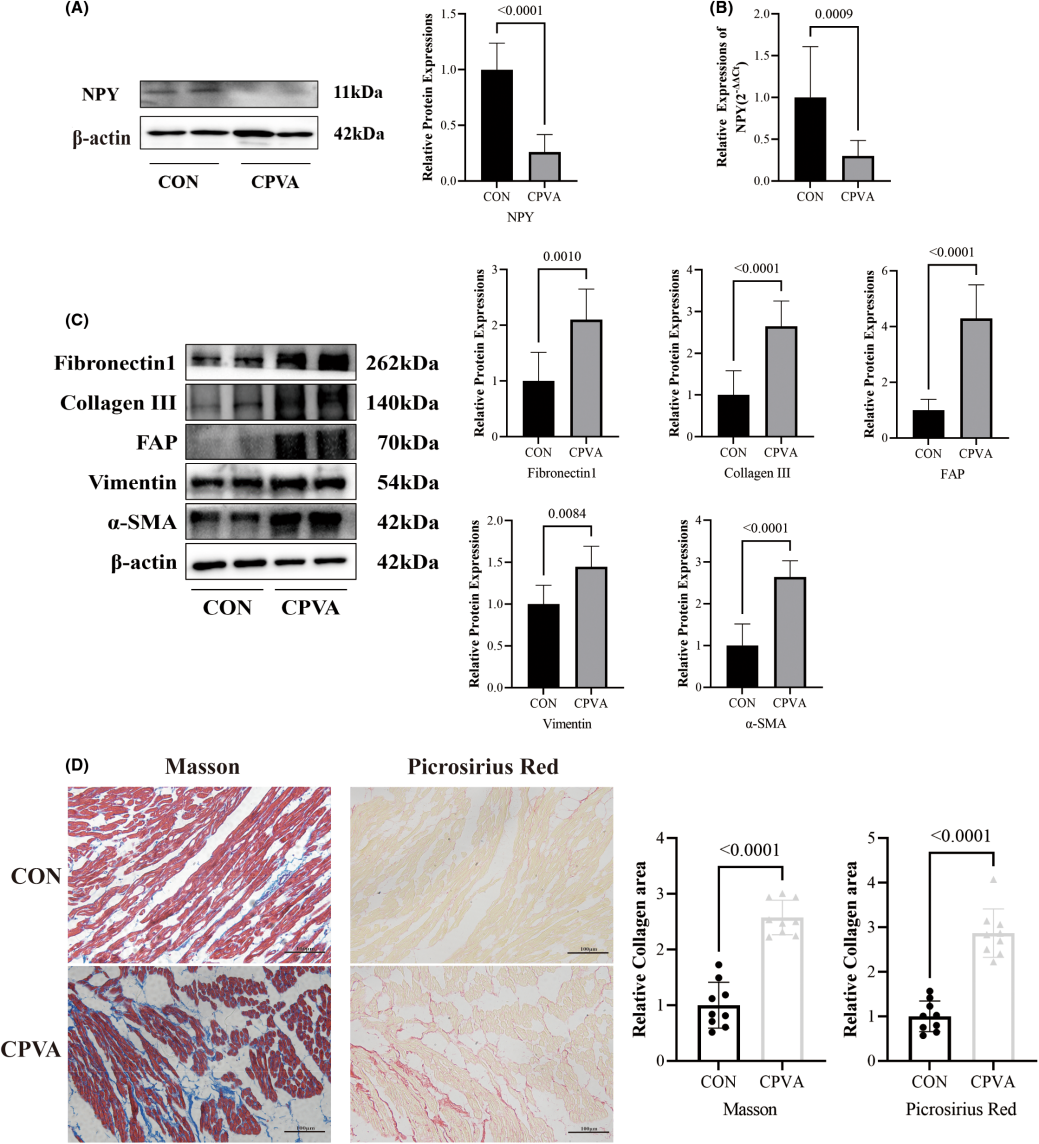
Changes in NPY expression and fibrosis degree in target atrial tissue 6 months after CPVA. (A) The relative expression of NPY in the control group and CPVA group was detected by Western blotting. (B) The relative expression of NPY in the control group and CPVA group was analysed by qRT–PCR. (C) The relative expression of Fibronectin1, Collagen III, FAP, Vimentin, α‐SMA in the control group and CPVA group was detected by Western blotting. (D) Masson staining and picrosirius red staining and relative quantitative analysis of collagen area percentage in target atrial tissue (*n* = 9, magnification 200×, scale bars show 100 μm).

Western blotting and q‐PCR results showed that NPY expression was reduced in the CPVA group compared with the control group in the long‐term (Figure 3A,B).

Western blotting results showed that the expression of Fibronectin1, Collagen III, FAP, Vimentin, α‐SMA was significantly higher in the target atrial muscle tissue of the CPVA group than that of the control group (Figure 3C). Masson staining and picrosirius red staining also showed that the degree of fibrosis was significantly higher in the target atrial muscle tissue of the CPVA group than that in the control group (Figure 3D). Therefore, we believe that fibrosis in the target atrial muscle tissue increased in the long‐term after CPVA, but the relationship between NPY and fibrosis remains elusive.

### 3.5 Neuropeptide Y can alleviate apoptosis of primary atrial myocytes and inhibit Akt activation of primary fibroblasts

**FIGURE 4:**
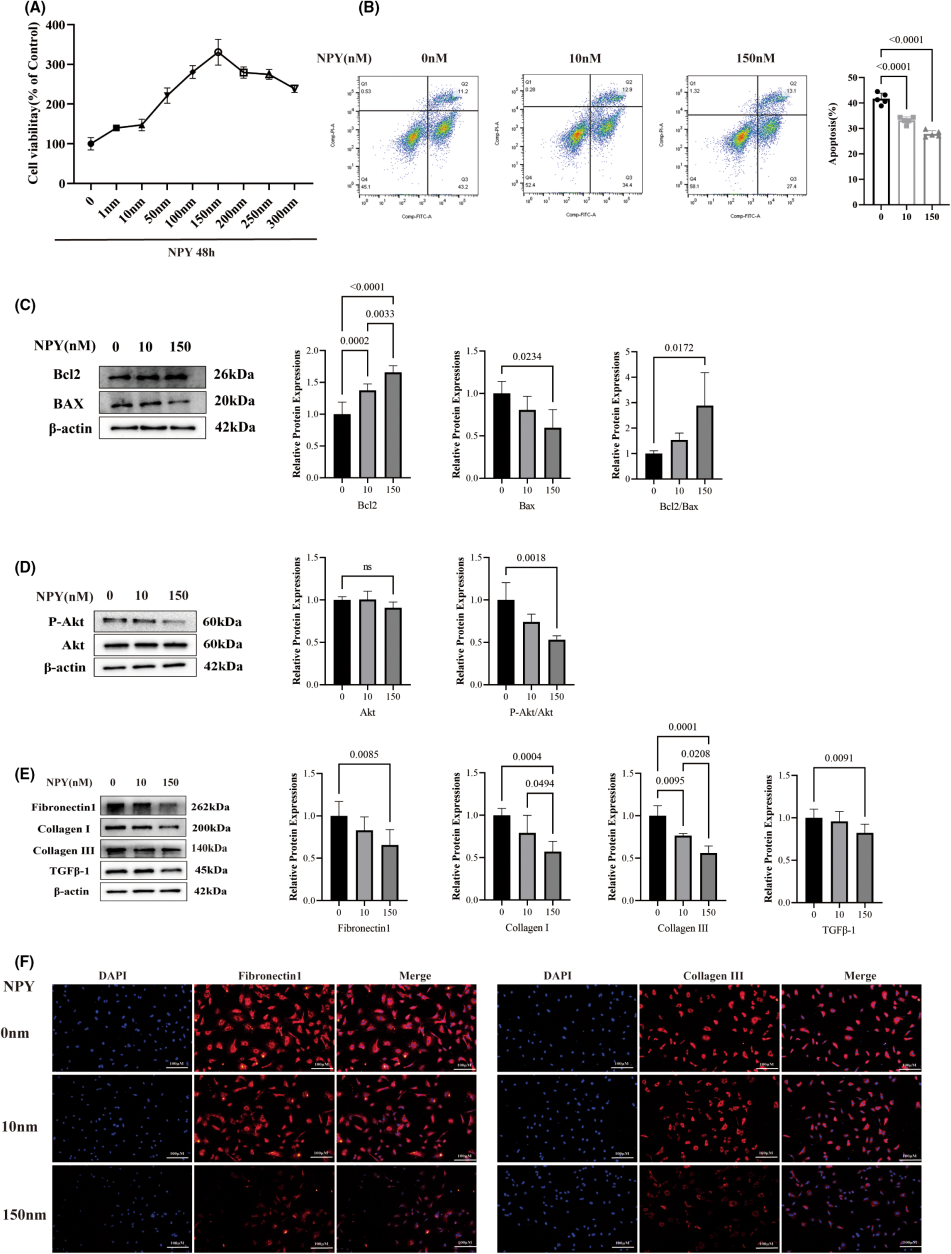
NPY alleviates apoptosis in primary rat atrial myocytes. (A) The CCK‐8 method was used for detecting the cell survival rate of the rat primary cells under the stimulation of different concentrations of NPY. (B) Flow cytometry was used to detect apoptosis and the quantitative percentage of primary cells stimulated by different concentrations of NPY. (C) The relative expression of Bcl‐2/Bax was detected by Western blot. (D) The relative expression of P‐Akt and Akt was detected by Western blot. NPY decreased the degree of fibrosis in rat primary cardiac fibroblasts and decreased Akt activation. (E) The relative expression of fibronectin 1, collagen I, collagen III and TGFβ‐1 was detected by western blotting. (F) The expression of fibronectin 1 and collagen III was detected by immunofluorescence.

Neuropeptide Y can alleviate the degree of apoptosis in primary rat atrial myocytes through the Bcl2‐BAX pathway. Primary atrial myocytes were treated with different concentrations of NPY, and CCK‐8 results showed that cell viability was best at 150 nM (Figure 4A). Therefore, the cells were divided into a control group, a low‐concentration NPY group (10 nM) and a high‐concentration NPY group (150 nM) and stimulated for 48 h. Flow cytometry showed that the apoptosis of primary rat atrial myocytes treated with 150 nM NPY was significantly lower than that of the control group (Figure 4B). Western blotting results showed that Bcl2/BAX was significantly increased in primary rat atrial myocytes treated with 0, 10 and 150 nM NPY, especially when treated with 150 nM (Figure 4C). Therefore, we suggest that NPY can alleviate the degree of apoptosis in rat primary atrial myocardium through the Bcl2‐Bax pathway. In addition, we found that the addition of NPY decreased the P‐Akt/Akt ratio in primary cardiac fibroblasts (Figure 4D).

### 3.6 Neuropeptide Y can inhibit the degree of fibrosis

Neuropeptide Y inhibits fibrosis in primary rat atrial fibroblasts. Western blotting showed that fibronectin 1, collagen I, collagen III and TGFβ‐1 were inhibited to different degrees in rat primary atrial fibroblasts treated with different concentrations of NPY (Figure 4E). By immunofluorescence staining, fibronectin 1 and collagen III showed the same trend (Figure 4F). Therefore, we suggest that NPY inhibits the degree of fibrosis in rat primary atrial fibroblasts, which may be related to its reduction in cardiomyocyte apoptosis and inhibition of Akt activation.

## 4 DISCUSSION

In this study, we found that the AERP of target atrial muscle tissue was shortened and fibrosis was increased after CPVA in the long‐term beagle canine model with CPVA. Transcriptome and proteomics shown differentially expressed genes, proteins and signalling pathway in the long‐term model of CPVA. In addition, NPY was found to be the most remarkably downregulated protein after CPVA. Moreover, NPY can alleviate the apoptosis of primary atrial myocytes and inhibit the activation of Akt in fibroblast and finally inhibit the fibrosis.

Atrial fibrillation is generally caused by ectopic activity, which usually originates from the PV.^19^, ^20^ CPVA can eliminates AF by blocking the conduction of ectopic activity from the PV to LA. However, AF recurrence still occurs frequently after successful PVI.^8^ These AF recurrence may be due to reconnection of PV‐LA or may be mediated by other arrhythmogenic factors present in various regions of the atrium.^21^, ^22^ A clinical study showed that PV reconnection was found in only 8.7% of patients with late gadolinium‐enhanced MRI used to assess the LA 3 months after ablation, specifically to assess the presence of continuous patchy scarring on the PVI line.^23^ Another clinical study^24^ showed that an invasive restudy of patients without AF recurrence for 1 year found restoration of PV conduction in 90.6% of patients, with similar incidence of PV reconnection in patients with and without AF recurrence. Early cicatricial PV reconnection does not fully explain the long‐term recurrence after CPVA. Our study found that beagle canines had a shortened left atrial AERP at 6 months after CPVA, which was more likely to induce AF in the long run, suggesting that some mechanism after CPVA causes AF reinduction.

In our study, we detected 31,897 different RNAs by high‐throughput RNA‐seq, of which 259 exhibited significant differences. The 4417 different proteins were detected by mass spectrometry, of which 186 exhibited significant differences. DEGs were more highly associated with inflammation, while DEPs were more highly associated with fibrosis and calcium transport. We suspect that this discrepancy may be due to two factors: on the one hand, there is a significant difference in detection thresholds between high‐throughput sequencing and mass spectrometry; on the other hand, there is post‐transcriptional regulation between mRNA and protein that has not been clarified.

Fibrosis was thought as the excessive deposition of extracellular matrix (ECM) proteins in parenchymal tissue, reflecting inappropriate or uncontrolled activation of repairing programmes.^25^, ^26^, ^27^ Myocardial fibrosis, including myocyte and collagen disorganization, ECM enlargement and left atrial fibrosis, has been reported to be independent factors for AF recurrence and a strong predictor of long‐term maintenance of sinus rhythm after AF ablation.^28^, ^29^ We found that the expression of collagen protein, including collagen I, collagen IV and collagen V, was significantly upregulated in the transcriptome and proteome of target atrial tissue after CPVA, and fibrosis‐related pathways, such as ECM‐receptor interactions and the PI3K‐Akt pathway, were enriched. The same results were obtained by fibrosis staining and quantitative analysis of related proteins in target atrial tissue, suggesting that atrial fibrosis may occur in long‐term ablation, which is a potential factor for AF reinduction.

Neuropeptide Y is the most downregulated DEP in our study, and it is the most abundant neuropeptide in the heart. However, it is rarely mentioned in AF studies. NPY is the pyramidal transmitter of norepinephrine in the sympathetic nerve and has effects on the whole cardiovascular system.^30^ NPY can be secreted from the sympathetic nerve endings of the heart and inhibits the release of acetylcholine from the parasympathetic nerve endings.^30^, ^31^, ^32^ NPY concentrations in normal human peripheral blood ranged from 90 to 140 pM.^33^ Tan et al.^9^ reported that adrenergic and cholinergic nerves were highly coaggregated within 5 mm of the PV‐LA junction, making highly selective ablation of vagus or sympathetic nerves impossible. Our previous study^16^ demonstrated that growth‐associated protein 43 (GAP43), tyrosine hydroxylase (TH) and choline acetyltransferase (CHAT) returned to preoperative levels at both 6 and 12 months after plexus ablation. These findings suggest that CA fails to maintain long‐term inhibition of cardiac autonomic nerves and that autonomic neuron remodelling will occur in the target atrial tissue late after ablation. Autonomic nerve redistribution and dysfunction may be the cause of NPY decrease in the long‐term after ablation.

Neuropeptide Y was reported to bind to receptors and play a role in cardiovascular diseases, including atherosclerotic ischaemia/infarction, arrhythmia, heart failure and hypertension.^30^, ^34^, ^35^ However, the role of NPY in cardiovascular disease was controversial, and many studies showed that the NPY had negative effects on cardiovascular system. It has been reported that plasma NPY levels are significantly elevated during myocardial infarction.^30^, ^36^ When sympathetic nerves are stimulated, NPY acts as a vasoconstrictor, causing chronic injury.^37^, ^38^ It had also been suggested that NPY was released when sympathetic nerve stimulation was a trigger for ventricular arrhythmia.^39^, ^40^ In contrast, in the myocardial infarction model, Qin et al.^41^ observed more severe myocardial infarction, higher mortality, progressive cardiac insufficiency, enlarged infarct size and increased cardiac inflammation and fibrosis in NPY‐deficient mice. Matyal et al.^42^ identified NPY as a potential therapeutic agent for apoptosis and fibrosis in a porcine model of chronic myocardial ischaemia. We speculated that the reason for the controversy may be that the effects of NPY on myocardium are concentration‐dependent. We treated primary rat atrial myocytes with appropriate concentrations of NPY, and apoptosis was alleviated. In addition, primary rat cardiac fibroblasts were treated with the same concentrations, and the degree of fibrosis and Akt activate were also alleviated. The role of the PI3K‐Akt signalling pathway in atrial fibrosis has been widely reported. Inhibition of PI3K‐Akt activation through different pathways has been observed to significantly improve atrial fibrosis.^43^, ^44^ Our previous study^45^, ^46^ showed that activation of the PI3K‐Akt signalling pathway in rats led to increased atrial fibrosis, which ultimately resulted in an increased rate of AF induction. Thus, we consider that NPY can inhibit fibrosis by alleviating the apoptosis of atrial myocytes and suppressing the activation of Akt in cardiac fibroblasts. However, the expression of NPY was decreased after CPVA, which led to the fibrosis of myocardium through the promotion of cardiomyocyte apoptosis and the activation of the PI3K‐Akt pathway in fibroblasts. Ultimately, this brings about the increased reinduction rate of AF in the long‐term.

Our study has some limitations. First, we did not deliberately observe the long‐term autonomic neuron remodelling by verifying GAP43, TH and CHAT because our previous studies and many other investigators had verified this phenomenon and the content of target atrial tissue is not enough. Second, we performed differential analysis only from different omics levels and coenriched pathways, focusing on the molecule NPY. In the future, we will explore other related targets and pathways and try to reveal the molecular mechanisms that may be related to AF reinduction. Third, we only studied the mechanism of NPY in apoptosis and fibrosis in vitro using primary rat atrial myocytes and didn't do interventional experiments in vivo. We hope to finish this work in the future study. Fourth, we lacked intervention on NPY receptors or on Akt activation to observe NPY ameliorating myocardial fibroblast fibrosis by inhibiting Akt activation and hope to test this mechanism in future studies.

## 5 CONCLUSIONS

In conclusion, our study suggests that the reinduction of long‐term AF in CPVA may be related to atrial fibrosis and the reduction in NPY. In vitro, we showed that NPY at appropriate concentrations can protect atrial myocytes from fibrosis by reducing cardiomyocyte apoptosis and inhibiting Akt activation in fibroblasts. This study provides novel insights for further study on the potential intervention targets of the AF recurrence mechanism.

## AUTHOR CONTRIBUTIONS

**Qiyuan Song:** Conceptualization (supporting); data curation (equal); methodology (equal); software (equal); validation (equal); visualization (equal); writing – original draft (equal); writing – review and editing (equal). **Ning Zhang:** Conceptualization (supporting); data curation (equal); methodology (equal); validation (equal). **Yujiao Zhang:** Data curation (equal); methodology (equal); software (equal); writing – review and editing (equal). **An Zhang:** Investigation (equal); software (equal). **Huilin Li:** Methodology (equal). **Shuting Bai:** Formal analysis (equal); visualization (equal). **Luxiang Shang:** Formal analysis (equal); visualization (equal); writing – review and editing (equal). **Juanjuan Du:** Conceptualization (supporting); formal analysis (equal); funding acquisition (equal); project administration (equal); resources (equal); supervision (equal); writing – review and editing (equal). **Yinglong Hou:** Conceptualization (lead); formal analysis (equal); funding acquisition (equal); project administration (equal); supervision (equal); writing – review and editing (equal).

## FUNDING INFORMATION

Natural Science Foundation of China (82100343), Shandong Province Natural Science Youth Foundation (ZR2020QH014 and ZR2022QH250), China Postdoctoral Science Foundation (2024M751869).

## CONFLICT OF INTEREST STATEMENT

The authors declare that the research was conducted in the absence of any commercial or financial relationships that could be construed as a potential conflict of interest.

## Supporting information


Table S1.

Table S2.

## Data Availability

The transcriptome data that support the findings of this study are openly available in GEO DataSets at https://www.ncbi.nlm.nih.gov/gds/, reference number GSE248285. The proteome data that support the findings of this study are openly available in iproX DataSets at https://www.iprox.cn/page/home.html, reference number PXD046888.
