# Supplementary material for: Multiomics analysis of canine myocardium after circumferential pulmonary vein ablation: Effect of neuropeptide Y on long‐term reinduction of atrial fibrillation
Source: J Cell Mol Med. 2024 Aug 6;28(15):e18582. doi: 10.1111/jcmm.18582 (PMC11303123; doi:10.1111/jcmm.18582)
Supplement: Supplementary file 1 — Table S1. [file JCMM-28-e18582-s002.docx]

**Supplementary Table 1：Top 20 DEGs**

| gene_id | gene_symbol | log2FoldChange | pvalue | padj |
| --- | --- | --- | --- | --- |
| ENSCAFG00000010505 | CCL20 | 23.2838 | 1.45E-14 | 2.01E-10 |
| ENSCAFG00000016447 | CD5L | 9.1767 | 3.09E-06 | 0.002664 |
| ENSCAFG00000013495 | OLR1 | 8.7743 | 3.05E-05 | 0.012228 |
| ENSCAFG00000004913 | MARCO | 8.4435 | 3.64E-05 | 0.012736 |
| ENSCAFG00000016261 | CSF3 | 8.149 | 6.84E-06 | 0.00472 |
| ENSCAFG00000046633 | CRB2 | 7.9504 | 0.000202 | 0.030683 |
| ENSCAFG00000024982 | C4BPA | 7.6125 | 6.05E-05 | 0.016407 |
| ENSCAFG00000007249 | IL1B | 7.5706 | 3.28E-05 | 0.012228 |
| ENSCAFG00000015205 | SAA1 | 7.4577 | 0.000148 | 0.026115 |
| ENSCAFG00000031174 | PPBP | 6.6637 | 1.45E-07 | 0.000391 |
| ENSCAFG00000013219 | BNC1 | 6.4079 | 0.001039 | 0.067979 |
| ENSCAFG00000031443 | EREG | 6.3468 | 2.32E-06 | 0.002211 |
| ENSCAFG00000013622 | IGLV2-33 | 6.265 | 0.01143 | 0.24242 |
| ENSCAFG00000010206 | CTSE | 6.2633 | 4.16E-06 | 0.003478 |
| ENSCAFG00000002142 | IL1RL1 | 6.1824 | 3.26E-05 | 0.012228 |
| ENSCAFG00000007419 | DNASE1L3 | 6.1544 | 9.91E-06 | 0.005913 |
| ENSCAFG00000007245 | IL1A | 6.0549 | 0.000218 | 0.032107 |
| ENSCAFG00000012211 | NDST4 | 5.8564 | 0.000294 | 0.036025 |
| ENSCAFG00000015054 | MMP1 | 5.8344 | 0.000364 | 0.039855 |
| ENSCAFG00000008936 | SPOCK3 | 5.8226 | 0.000884 | 0.062772 |
