# Supplementary material for: Multiomics analysis of canine myocardium after circumferential pulmonary vein ablation: Effect of neuropeptide Y on long‐term reinduction of atrial fibrillation
Source: J Cell Mol Med. 2024 Aug 6;28(15):e18582. doi: 10.1111/jcmm.18582 (PMC11303123; doi:10.1111/jcmm.18582)
Supplement: Supplementary file 2 — Table S2. [file JCMM-28-e18582-s001.docx]

**Supplementary Table 2：Top 20 DEPs**

| gene_id | gene_symbol | FoldChange | pvalue | log2FoldChange |
| --- | --- | --- | --- | --- |
| ENSCAFP00000004135 | NPY | 0.662221 | 0.024601 | -0.59462 |
| ENSCAFP00000053222 | VCAN | 2.094833 | 0.00492 | 1.066835 |
| ENSCAFP00000022374 | JAK3 | 1.957985 | 0.033243 | 0.96937 |
| ENSCAFP00000050585 | RBPJ | 1.902817 | 0.014361 | 0.928137 |
| ENSCAFP00000013230 | THBS4 | 1.894949 | 0.032085 | 0.922159 |
| ENSCAFP00000043876 | UPRT | 1.768067 | 0.020844 | 0.822173 |
| ENSCAFP00000023268 | PADI2 | 1.739087 | 0.032774 | 0.79833 |
| ENSCAFP00000017808 | CTSK | 1.699283 | 0.01008 | 0.764926 |
| ENSCAFP00000006879 | CLPTM1 | 1.697057 | 0.009199 | 0.763035 |
| ENSCAFP00000047900 | IFIT2 | 1.684599 | 0.035498 | 0.752406 |
| ENSCAFP00000009210 | KERA | 1.680641 | 0.043301 | 0.749012 |
| ENSCAFP00000049612 | PCLO | 1.670925 | 0.018659 | 0.740647 |
| ENSCAFP00000003355 | OGN | 1.654618 | 0.047303 | 0.726498 |
| ENSCAFP00000048085 | COL5A2 | 1.608166 | 0.042829 | 0.685417 |
| ENSCAFP00000058078 | TSPAN11 | 1.579044 | 0.036729 | 0.659051 |
| ENSCAFP00000050714 | C7 | 1.575364 | 0.003266 | 0.655685 |
| ENSCAFP00000051570 | COL14A1 | 1.573916 | 0.034725 | 0.654358 |
| ENSCAFP00000004489 | AFP | 1.532327 | 0.019488 | 0.615724 |
| ENSCAFP00000028297 | BGN | 1.512534 | 0.017801 | 0.596968 |
| ENSCAFP00000009637 | APBB1 | 1.498142 | 0.008249 | 0.583174 |
